# Left Ventricular Remodeling and Myocardial Work: Results From the Population-Based STAAB Cohort Study

**DOI:** 10.3389/fcvm.2021.669335

**Published:** 2021-06-11

**Authors:** Floran Sahiti, Caroline Morbach, Vladimir Cejka, Judith Albert, Felizitas A. Eichner, Götz Gelbrich, Peter U. Heuschmann, Stefan Störk

**Affiliations:** ^1^Comprehensive Heart Failure Center, University and University Hospital Würzburg, Würzburg, Germany; ^2^Department of Medicine I, University Hospital Würzburg, Würzburg, Germany; ^3^Institute of Clinical Epidemiology and Biometry, University of Würzburg, Würzburg, Germany; ^4^Clinical Trial Center, University Hospital Würzburg, Würzburg, Germany

**Keywords:** myocardial work, myocardial work efficiency, left ventricular geometry, left ventricular mass, LV dilatation, left ventricular geometric abnormality, left ventricular remodeling

## Abstract

**Introduction:** Left ventricular (LV) dilatation and LV hypertrophy are acknowledged precursors of myocardial dysfunction and ultimately of heart failure, but the implications of abnormal LV geometry on myocardial function are not well-understood. Non-invasive LV myocardial work (MyW) assessment based on echocardiography-derived pressure-strain loops offers the opportunity to study detailed myocardial function in larger cohorts. We aimed to assess the relationship of LV geometry with MyW indices in general population free from heart failure.

**Methods and Results:** We report cross-sectional baseline data from the Characteristics and Course of Heart Failure Stages A-B and Determinants of Progression (STAAB) cohort study investigating a representative sample of the general population of Würzburg, Germany, aged 30–79 years. MyW analysis was performed in 1,926 individuals who were in sinus rhythm and free from valvular disease (49.3% female, 54 ± 12 years). In multivariable regression, higher LV volume was associated with higher global wasted work (GWW) (+0.5 mmHg% per mL/m^2^, *p* < 0.001) and lower global work efficiency (GWE) (−0.02% per mL/m^2^, *p* < 0.01), while higher LV mass was associated with higher GWW (+0.45 mmHg% per g/m^2^, *p* < 0.001) and global constructive work (GCW) (+2.05 mmHg% per g/m^2^, *p* < 0.01) and lower GWE (−0.015% per g/m^2^, *p* < 0.001). This was dominated by the blood pressure level and also observed in participants with normal LV geometry and concomitant hypertension.

**Conclusion:** Abnormal LV geometric profiles were associated with a higher amount of wasted work, which translated into reduced work efficiency. The pattern of a disproportionate increase in GWW with higher LV mass might be an early sign of hypertensive heart disease.

## Introduction

The constant exposure to cardiovascular risk factors and/or adverse hemodynamic conditions induces complex changes in left ventricular (LV) geometry, often starting as a physiological compensatory response (1, 2). Alterations in LV geometry such as LV dilatation and LV hypertrophy are acknowledged precursors of myocardial dysfunction and ultimately of heart failure (3–6), but the mechanisms are still not well-understood. Invasive recording of pressure-volume loops as the reference standard provides real-time assessment of LV loading conditions, contractility, and myocardial oxygen consumption (7). However, its (repeated) use in clinical routine is limited due to the investigation's invasive nature. Recent advances in imaging methods allow to approximate the intrinsic and functional cardiac performance with satisfactory precision, also accounting for loading conditions. A novel echocardiographic method has been introduced and validated against invasive measurements that non-invasively quantifies active myocardial function, i.e., systolic and early diastolic active myocardial work (MyW) (8). This approach allows differentiating constructive from wasted MyW, with the latter not contributing to LV output. The concept of MyW measurement is based on speckle-tracking derived longitudinal strain and systolic blood pressure and is widely applicable, including situations of screening. However, echocardiography-derived MyW has to be differentiated from the puristic definition of cardiac work derived from invasive pressure-volume loops, expressed in Joule or Centijoule (9). MyW approximates the work contributing to LV output, i.e., constructive work, and quantifies energy loss due to uncoordinated left ventricular contractions resulting in stretching of individual LV segments by the contraction of other LV segments, i.e., wasted work (10). Further, MyW might allow profound insights into LV performance and, given the strong correlation with cardiac glucose uptake as measured by positron emission tomography, might also serve as surrogate of regional and global myocardial metabolism (8, 10). LV geometry patterns have been shown to be of prognostic relevance in community studies (11, 12) and depend, i.e., on exposure to modifiable cardiovascular risk factors, such as hypertension and obesity (4, 13, 14). Thus, the detailed evaluation of MyW in relation to LV geometry might further advance the pathophysiological understanding of functional changes associated with abnormal LV geometry. Therefore, we aimed to assess the association of LV geometry with myocardial work in a well-characterized population-based sample of individuals free from heart failure.

## Methods

### Population

Within the Characteristics and Course of Heart Failure STAges A/B and Determinants of Progression (STAAB) prospective cohort study, we recruited and comprehensively phenotyped a representative sample of the population of Würzburg, Germany, aged 30–79 years, *n* = 5,000, free of symptomatic heart failure. The study design and baseline characteristics have been published previously (15, 16). The STAAB study complies with the Declaration of Helsinki and was approved by the ethics committee, University of Würzburg (J-117.605-09/13). All participants provided written informed consent prior to any study-related examination. For the present analysis, we evaluated cross-sectional data of the baseline examination from the first half of the STAAB study population (*n* = 2,473). This group had been included between December 12, 2013, and September 2, 2016, was pre-specified for a planned interim analysis (15), and therefore met the sex and age stratification criteria of the total sample.

### Baseline Examination

Participants were evaluated at the Joint Survey Unit of the Comprehensive Heart Failure Center and the Institute for Clinical Epidemiology and Biometry, University of Würzburg. Routine laboratory measurements were performed at the central laboratory of the University Hospital Würzburg, including fasting lipid profile, estimated glomerular filtration rate (eGFR), glycosylated hemoglobin (HbA1c), and NT-proBNP levels. Blood pressure (in a sitting position after 5 min of rest), body height and weight, hypertension history, and current anti-hypertensive pharmacotherapy were assessed according to standard operating procedures (14). According to ESC guidelines, the presence of hypertension was defined as blood pressure ≥140/90 mmHg or on anti-hypertensive pharmacotherapy (17). We further sub-classified our sample according to blood pressure into four groups as recommended by current guidelines (17): (a) optimal blood pressure, i.e., systolic blood pressure (SBP) <120 mmHg; (b) normal blood pressure, SBP 120–129 mmHg; (c) high-normal blood pressure, SBP 130–139 mmHg; and (d) grade 1 hypertension or higher, SBP ≥140 mmHg.

### Echocardiographic Analysis and LV Geometry

**Figure 1:**
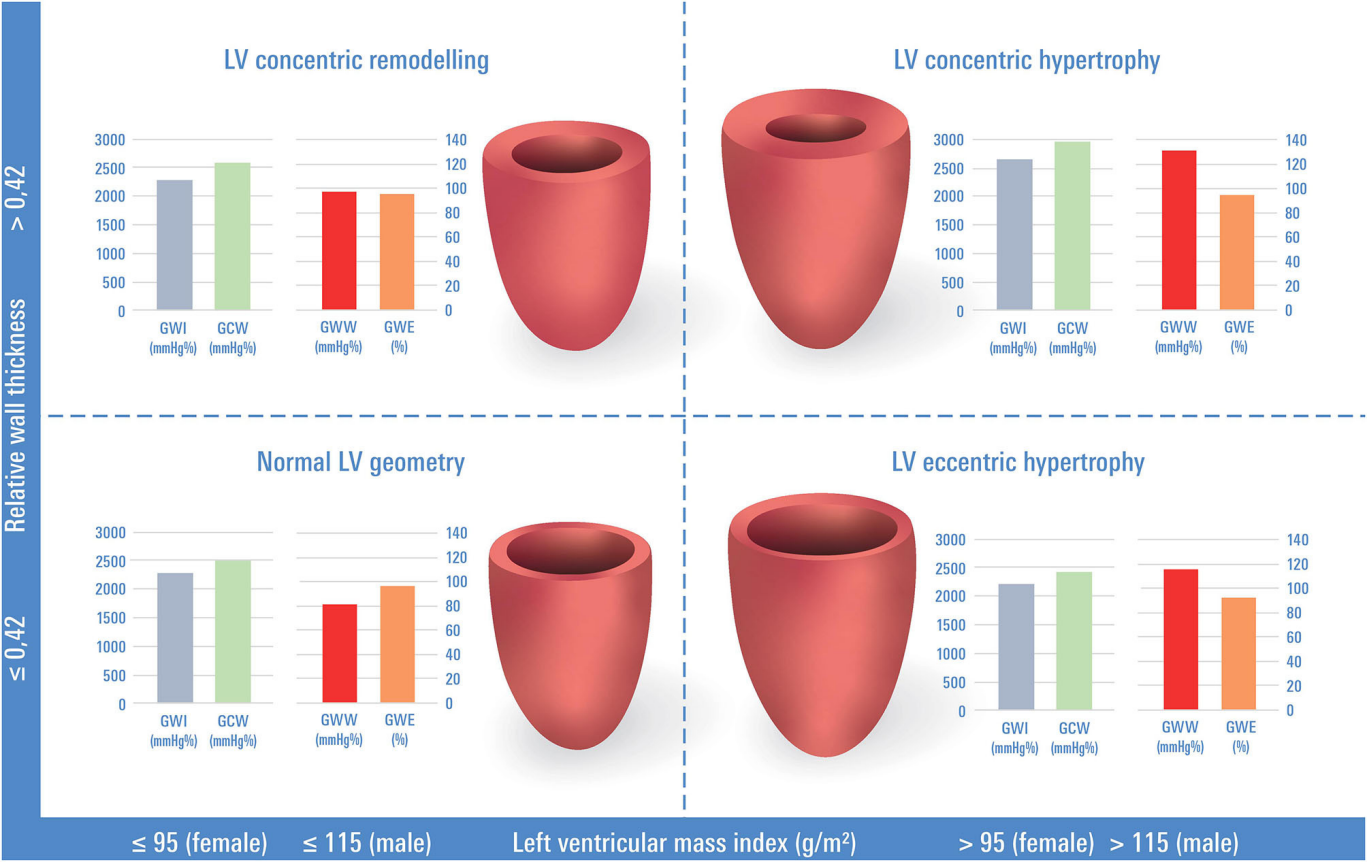
Classification of left ventricular geometry based on the left ventricular mass index and relative wall thickness and myocardial work indices. Columns with myocardial work indices represent the mean or median values of our study sample. GWI, global work index; GCW, global constructive work; GWW, global wasted work, GWE, global work efficiency.

Image acquisition was performed by trained and certified sonographers employing one echocardiography machine (Vivid S6® with M4S Sector Array Transducer operating at 1.5–4.3 MHz, GE Healthcare, Horten, Norway) with presets maintained according to a pre-specified protocol. The utility of performance measures of the echocardiography quality assurance program has been published previously (18). A minimum of three cardiac cycles was recorded. Two-dimensional images from the LV apical four-, two-, and three-chamber views were recorded with a frame rate of 50–80 s^−1^ and stored digitally. We derived end-diastolic interventricular septum thickness (IVSd), LV posterior wall thickness (LVPWd), and LV end-diastolic diameter (LVEDD) in the parasternal long-axis from an M-Mode recording, or—in case of suboptimal angulation—from a 2D measurement (19). We calculated LV mass using the corrected American Society of Echocardiography method (19): LV mass (g) = 0.8 (1.04 [([LVEDD + IVSd +LVPWd]^3^ - LVEDD^3^)]) + 0.6 as well. LV relative wall thickness (RWT) was calculated as: (2 ^*^ posterior wall thickness)/LV end-diastolic diameter (1, 19). We further measured LV ejection fraction (LVEF) and LV end-diastolic volume using Simpson's biplane method (19). Early diastolic myocardial relaxation velocity (e') was assessed using tissue and PW-Doppler close to the septal and/or lateral mitral annulus. LA volume was measured biplane in apical four and two-chamber view and left atrial volume index (LAVi) was calculated as LA volume indexed to body surface area. Valve regurgitation was determined by the color Doppler multiplane vena contracta method, and valve stenosis was quantified by continuous-wave Doppler measurements (15). LV mass index (LVMi) and LV end-diastolic volume index (LVEDVi) were calculated, indexing LV mass and LV end-diastolic volume to body surface area, respectively. According to the latest guidelines (1, 19), we classified the participants into four different subgroups according to their respective LV geometry pattern (Figure 1): (a) normal LV geometry, LVMi ≤95 g/m^2^ in women or ≤115 g/m^2^ in men and RWT ≤0.42; (b) concentric LV remodeling (CR), LVMi ≤95 g/m^2^ in women or ≤115 g/m^2^ in men and RWT >0.42; (c) concentric LV hypertrophy (CH), LVMi >95 g/m^2^ in women or >115 g/m^2^ in men and RWT >0.42; (d) eccentric LV hypertrophy (EH), LVMi >95 g/m^2^ in women or >115 g/m^2^ in men and RWT ≤0.42.

### Myocardial Work Analysis

MyW analysis was performed off-line based on the stored echocardiography images and blood pressure measurements. Aortic and mitral valve closure and opening times were assessed by CW Doppler of the aortic valve and PW Doppler of the mitral valve. However, as potential changes in heart rate during the examination might affect the loop area, these time points were visually verified in the apical three-chamber view and manually adjusted where necessary. LV apical four-, two-, and three-chamber views were analyzed off-line using Automated Functional Imaging (EchoPAC®, Version 202, GE) to determine global longitudinal strain (GLS). Provision of peripheral blood pressure allowed the derivation of the MyW parameters as detailed by others (8, 10, 20).

A) Global constructive work [GCW (mmHg%)], i.e., the sum of positive work performed during shortening in systole and adding negative work during lengthening in isovolumic relaxation;
B) Global wasted work [GWW (mmHg%)], i.e., the sum of negative work performed during lengthening in systole plus work performed during shortening against a closed aortic valve in isovolumic relaxation;
C) Global work index [GWI (mmHg%)], i.e., the total work performed from mitral valve closure to mitral valve opening.
D) Global work efficiency [GWE (%)], i.e., GWE= GCW / (GCW + GWW).

**Figure 2:**
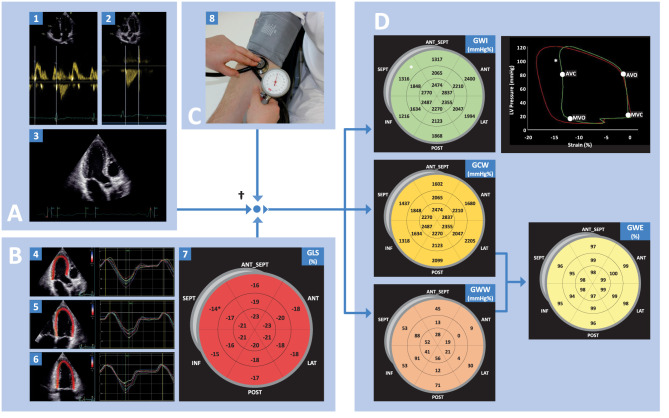
Illustrative physiological background of LV myocardial work analysis. **(A)** Represents valvular times, with mitral valve opening and close measured using pulse-waved Doppler derived mitral inflow and aortic valve opening and closure measured by continuous-wave Doppler derived transaortic outflow. **(B)** Global longitudinal strain measured from 4, 3, and 2 chamber view. **(C)** Estimated LV Pressure measured from brachial cuff pressure. **(D)** Schematic presentation of segment-specific values of MyW indices, which later are expressed in global values. GCW and GWW are important physiological indices related to the shortening and lengthening of the LV segments. Work efficiency (GWE) is derived as the fraction of GCW and the sum of GCW and GWW. ^†^ Empiric reference curve of LV pressure as suggested in the validation study by Russell et al. (8). * indicates a segment-specific pressure-strain loop (in this case, we highlighted the septal basal segment).

All indices were calculated as the mean of respective segmental values (18-segment model). We excluded subjects from further analysis in whom >1 LV segment was unsuitable for analysis due to poor tracking or suboptimal image quality. Determination of MyW, as well as quality assurance measures, have been published previously (21). Figure 2 illustrates step by step the approach to LV myocardial work analysis.

### Data Analysis

Continuous variables are described as mean (standard deviation) and categorical variables as frequency (percent). Normal distribution was checked using the Shapiro-Wilk test. Normal distributed variables were compared using the *t*-test, non-normal distributed variables using the Mann-Whitney *U*-test, and categorical variables using the chi-square test, respectively. Differences between groups were tested using the Kruskal-Wallis test, median test, and chi-square test. To test the relationship between LV geometry and MyW, we first ran a univariable linear regression analysis for each of MyW indices. Because we wanted to describe the relative contribution of systolic blood pressure, this variable was also tested, despite the fact that it is part of the derivation of myocardial work indices. In subsequent multivariable models, however, systolic blood pressure was omitted. Models were based on results of univariable regression and their physiological context. Thus, the multivariable model included age, sex, body mass index (BMI), LVEF, GLS, heart rate, low-density lipoprotein (LDL), glycosylated hemoglobin (HbA1c), hypertension, and measures of LV geometry such as LVMi and LVEDVi. The Jonckheere-Terpstra test was used for trend analysis. All tests were performed 2-sided. *P*-value < 0.05 were considered statistically significant. Statistical analysis was performed using SPSS (Version 26, SPSS Inc., Chicago, USA).

## Results

**Figure 3:**
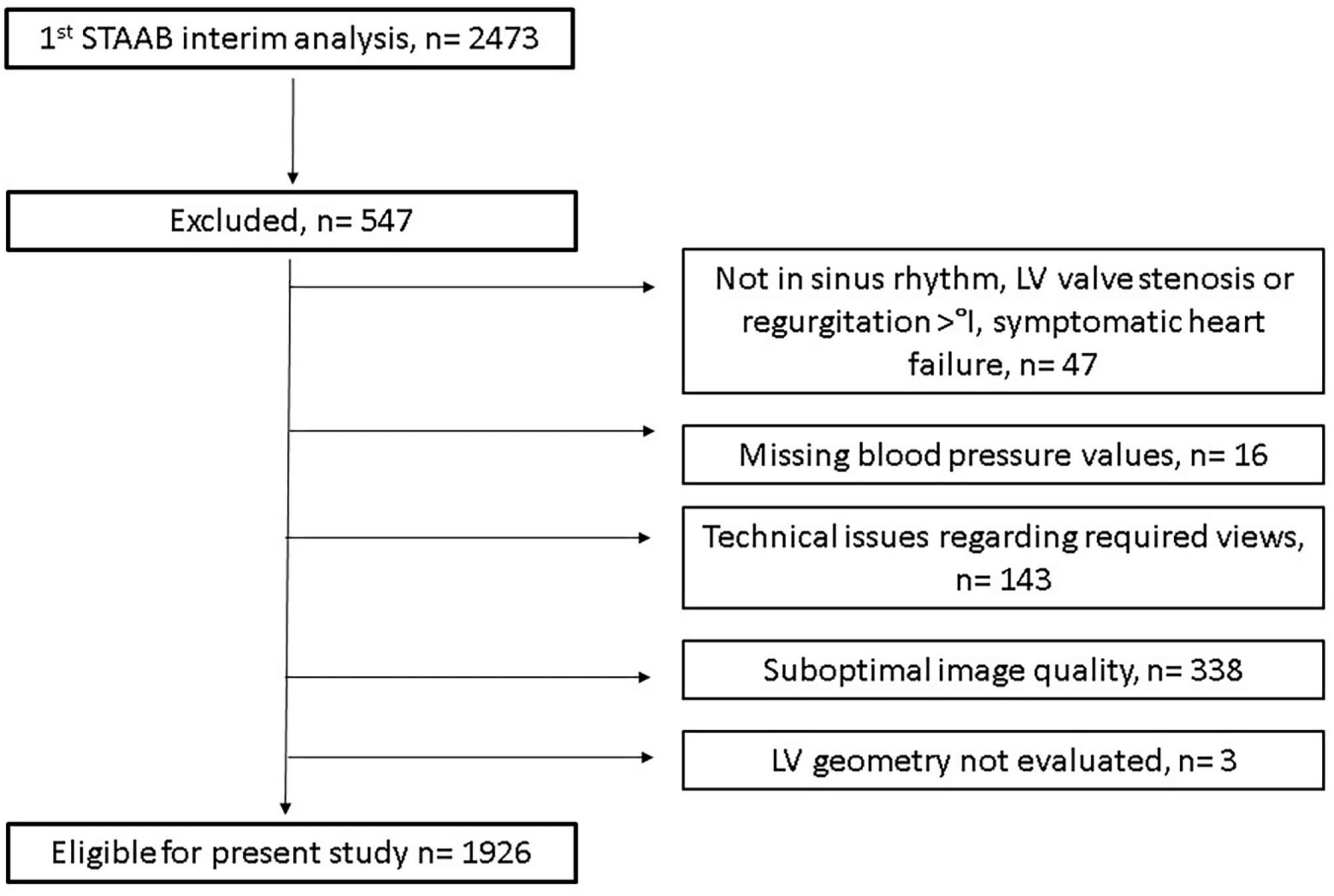
Study flow.

For the pre-planned interim analysis of the STAAB cohort study, 2,473 individuals were considered. Of those, a total of *n* = 547 participants were excluded from the current analysis for different reasons including technical issues regarding required views, poor tracking or suboptimal image quality, or missing blood pressure values (for details, see Figure 3). Therefore, a total sample of *n* = 1,926 individuals was included (49.3% women, with mean age 54 ± 12 years). Ninety-three percent of those had normal LV geometry, and 5% exhibited CR, 2% had EH, and <1% had CH, respectively. Table 1 presents the clinical and echocardiographic characteristics for the total sample and stratified for groups defined by LV geometry.

**Table 1:** Baseline characteristics in the total sample and according to left ventricular (LV) geometry.

	**All subjects (*N* = 1,926)**	**LV normal geometry (*N* = 1,789)**	**LV concentric remodeling (*N* = 100)**	**LV concentric hypertrophy (*N* = 6)**	**LV eccentric hypertrophy (*N* = 31)**
Age [years]	54 (12)	53 (12)	61 (10)^*^	69 (16)^*^	61 (9)^*^
Sex, women	950 (49.3)	879 (49.1)	48 (48.0)	4 (66.6)	19 (61.2)
BSA [m^2^]	1.9 (0.2)	1.9 (0.2)	1.9 (0.2)	1.9 (0.3)	1.9 (0.2)
BMI [kg/m^2^]	26.0 (4.3)	25.9 (4.1)	28.3 (4.7)^*^	30.1 (9.0)	28.1 (5.0)^*^
Heart rate [beats/min]	67 (10)	67 (10)	69 (10)^*^	60 (5)^*^	65 (12)
SBP [mmHg]	130 (18)	130 (17)	141 (18)^*^	148 (12)^*^	139 (23)^*^
DBP [mmHg]	78 (10)	78 (10)	81 (8)^*^	79 (13)	78 (14)
NT-proBNP [pg/ml]	52 (24, 97)	51 (24, 94)	52 (29, 108)	87 (63, 245)	154 (58, 305)^*^
LDL cholesterol [mg/dl]	122 (34)	122 (34)	126 (34)^*^	113 (27)	124 (44)^*^
HbA1c [%]	5.5 (0.6)	5.5 (0.5)	5.9 (1.0)^*^	5.8 (0.5)	6.1 (1.1)^*^
eGFR [ml/min]	87 (15)	87 (15)	83 (15)	85 (19)	86 (17)
Hypertension	848 (44.0)	735 (41.1)	79 (79.0)^*^	6 (100)^*^	28 (90.3)^*^
Diabetes	155 (8.0)	124 (6.9)	20 (20.0)^*^	3 (50.0)^*^	8 (25.8)^*^
Obesity	301 (15.6253)	253 (14.1)	33 (33.0)^*^	2 (33.3)	13 (41.9)^*^
Dyslipidemia	254 (13.2)	222 (12.4)	19 (19.0)	2 (33.3)	11 (35.5)^*^
Coronary heart disease	70 (3.6)	53 (2.9)	8 (8.0)^*^	1 (16.6)	8 (25.8)^*^
Peripheral artery disease	25 (1.3)	21 (1.2)	2 (2.0)	0 (0)	2 (6.5)^*^
Anti-hypertensive therapy	522 (27.1)	436 (24.4)	59 (59.0)^*^	5 (83.3)^*^	22 (70.9)^*^
ACEi/ARB	382 (19.8)	318 (17.7)	45 (45.0)^*^	5 (83.3)^*^	14 (45.2)^*^
Beta-blocker	242 (12.6)	200 (11.2)	26 (26.0)^*^	3 (50.0)^*^	13 (41.9)^*^
Diuretics	99 (5.1)	81 (4.5)	11 (11.0)^*^	2 (33.3)^*^	5 (16.1)^*^

* *Explorative comparison with individuals with normal LV geometry (two-sided p < 0.05)*.

*Data are n (%), mean (SD), or median (quartiles)*.

*BSA, body surface area; BMI, body mass index; NT-proBNP, N-terminal-pro Brain Natriuretic Peptide; SBP, systolic blood pressure; DBP, diastolic blood pressure; LDL, low-density lipoprotein; HbA1c, glycosylated hemoglobin; eGFR, estimated glomerular filtration rate, ACEi, angiotensin converting enzyme inhibitor, ARB, angiotensin II receptor type 1 blocker. Medications history was obtained in n = 1,914 individuals*.

Participants with normal LV geometry were younger and had lower BMI, SBP, NT-proBNP, LDL cholesterol, and HbA1c compared to abnormal geometric LV patterns (Table 1). Accordingly, participants with normal LV geometry exhibited less often obesity, hypertension, diabetes mellitus, or dyslipidemia. In contrast, coronary heart disease and anti-hypertensive treatment was more prevalent in individuals with abnormal LV geometry patterns. Even though still within the normal range, LVEF and GLS were more favorable in normal LV geometry when compared to CR and EH (Table 2). LVEDV index was lower in CR and higher in EH participants. Diastolic function in abnormal LV geometry patterns was significantly less favorable when compared to normal LV geometry. MyW characteristics are shown in Table 2. When compared to normal LV geometry, we found higher values of GCW and GWI in CH, as well as of GWW in CR and EH. These effects resulted in compromised GWE with any type of abnormal LV geometry.

**Table 2:** Baseline echocardiographic characteristics including myocardial work according to the LV geometry classification.

	**All subjects (*N* = 1,926)**	**LV normal geometry (*N* = 1,789)**	**LV concentric remodeling (*N* = 100)**	**LV concentric hypertrophy (*N* = 6)**	**LV eccentric hypertrophy (*N* = 31)**
IVSd [mm]	9 (1)	9 (1)	10 (1)^*^	11 (1)^*^	9 (1)^*^
LVPWd [mm]	8 (1)	8 (1)	10 (1)^*^	11 (1)^*^	11 (1)^*^
LVEDd [mm]	48 (5)	48 (5)	44 (4)^*^	51 (4)	55 (4)^*^
RWT	0.34 (0.05)	0.33 (0.05)	0.45 (0.04)^*^	0.44 (0.02)^*^	0.35 (0.04)^*^
LVM [g]	138 (39)	136 (37)	153 (36)^*^	219 (43)^*^	219 (42)^*^
LVMi [g/m^2^]	72 (16)	71 (15)	78 (15)^*^	113 (13)^*^	112 (10)^*^
LVEDV [mL]	99 (25)	99 (25)	93 (22)^*^	100 (33)	123 (29)^*^
LVEDVi [mL/m^2^]	52 (10)	52 (10)	47 (9)^*^	52 (16)	64 (14)^*^
LAV [mL]	46 (15)	46 (15)	47 (16)	54 (12)	55 (17)^*^
LAVi [mL/m^2^]	24 (7)	24 (7)	25 (8)	28 (7)	29 (9)^*^
E prime lateral	11 (3)	11 (3)	9 (2)^*^	7 (2)^*^	8 (3)^*^
E prime septal	9 (2)	9 (2)	7 (2)^*^	5 (1)^*^	6 (2)^*^
LVEF [%]	61 (4)	61 (4)	60 (4)^*^	59 (3)	58 (7)^*^
Stroke volume [ml]	60 (15)	60 (15)	55 (14)^*^	58 (16)	70 (16)^*^
GLS [–%]	21 (3)	21 (3)	20 (2)^*^	21 (1)	19 (3)^*^
GCW [mmHg%]	2,506 (428)	2,501 (424)	2,575 (457)	2,965 (240)^*^	2,445 (526)
GWW [mmHg%]	83 (59, 119)	81 (58, 118)	98 (68, 133)^*^	130 (80, 191)	117 (90, 158)^*^
GWI [mmHg%]	2,278 (396)	2,276 (392)	2,311 (424)	2,670 (315)^*^	2,207 (502)
GWE [%]	96 (95, 97)	96 (95, 97)	95 (94, 97)^*^	94 (91, 96)	94 (93, 95)^*^

* *Significantly different when compared to LV normal geometry (two-sided p < 0.05)*.

*Data are n (%), mean (SD), or median (quartiles)*.

*LVEF, left ventricular ejection fraction; GLS, global longitudinal strain, IVSd, interventricular septum diameter; LVPWd, left ventricular posterior wall diameter; LVEDd, left ventricular end-diastolic diameter; RWT, relative wall thickness; LVM, left ventricular mass; LVMi, left ventricular mass index; LVEDV, left ventricular end-diastolic volume; LVEDVi, left ventricular end-diastolic volume index; LAV, left atrial volume; LAVi, left atrial volume index, GCW, global constructive work; GWW, global wasted work; GWI, global work index; GWE, global work efficiency*.

In multivariable linear regression analysis including age, sex, BMI, heart rate, LVEF, LDL, HbA1c, hypertension, LVMi, and LVEDVi, we found that higher LV muscle mass was associated with a higher GCW, but also with higher GWW, thus resulting in reduced GWE. In contrast, higher LV volume was associated with higher GWW only, which also resulted in lower GWE (Table 3). In a further step, we analyzed patients with normal LV geometry according to the presence of hypertension (Table 4). Individuals with hypertension were more often male, were older, and had higher BSA and BMI. They showed similar LV volumes but significantly higher LV mass and LA volume and less favorable measures of systolic and diastolic function. Individuals with hypertension revealed significantly higher GCW and GWI, but also GWW, resulting in lower GWE. A sensitivity analysis focusing on the current blood pressure category showed a consistent pattern, i.e., higher GCW, GWI, and GWW with increasing blood pressure but lower GWE (Table 5). The strength of the association for the trends observed in Tables 4, 5 was maintained when adjusting for age.

**Table 3:** Univariable and multivariable regression analysis of myocardial work indices and different echocardiographic parameters.

	**GCW [mmHg%]**		**GWW [mmHg%]**		**GWI [mmHg%]**		**GWE [%]**	
	**Mean 2,506, SD 428**		**Median 83, quartiles 59, 119**		**Mean 2,278, SD 396**		**Mean 96, SD 2**	
	**Univariable analysis**	**Multivariable analysis^†^**	**Univariable analysis**	**Multivariable analysis^†^**	**Univariable analysis**	**Multivariable analysis^†^**	**Univariable analysis**	**Multivariable analysis^†^**
Sex [Women]	+87.3^***^	ns	−0.5	+10.9^***^	+124^***^	+68.8^***^	+0.2^*^	−0.4^***^
Age [years]	+7.9^***^	+4.5^***^	+1.2^***^	+0.8^***^	+4.9^***^	+1.6^*^	−0.05^***^	−0.03^***^
BMI [kg/m^2^]	−6.5^**^	−10.1^***^	+0.3	−1.3^***^	−4.9^*^	−5.7^**^	−0.02^*^	+0.05^***^
LVEF [%]	+19.4^***^	+11.0^***^	−2.1^***^	−1.4^***^	+21.9^***^	+13.1^***^	+0.1^***^	+0.08^***^
GLS [–%]	+50.4^***^	+51.4^***^	−2.5^***^	−1.2^**^	+50.9^***^	+48.4^***^	+0.2^***^	+0.1^***^
Heart rate [beats/min]	−4.9^***^	ns	+0.3^**^	+0.4^***^	−5.5^***^	−2.6^**^	−0.02^***^	−0.02^***^
Systolic BP [mmHg]	+16.6^***^	–	+1.1^***^	–	+14.3^***^	–	−0.02^***^	–
LDL-C [mg/dl]	+0.3	ns	+0.06	ns	+0.2	ns	−0.002	ns
HbA1c [%]	+10.5	−35.7^*^	+10.6^***^	ns	−1.7	ns	−0.5^***^	ns
LVEDVi [mL/m^2^]	−2.0^*^	ns	+0.3^**^	+0.5^***^	−2.0^*^	ns	−0.02^***^	−0.02^***^
LVMi [g/m^2^]	+2.4^***^	+2.0^**^	+0.7^***^	+0.4^***^	+1.1^*^	+1.5^**^	−0.03^***^	−0.01^***^
IVSd [mm]	+21.9^**^	–	+6.7^***^	–	+10.6	–	−0.3^***^	–
LVPWd [mm]	+16.6^*^	–	+6.1^***^	–	+4.3	–	−0.3^***^	–
LVEDd [mm]	−3.1	–	+0.6^*^	–	−4.8^*^	–	−0.03^**^	–
RWT	+539^**^	–	+109^***^	–	+351^*^	–	−4.5^***^	–
Hypertension	+304^***^	+343^***^	+29.2^***^	+19.4^***^	+253^***^	+316^***^	−0.9^***^	−0.3^**^

*(–) indicates that the variable was not considered in the multivariable regression analysis*.

*GCW, global constructive work, GWW, global wasted work; GWI, global work index; GWE, global work efficiency; BMI, body mass index; LVEF, left ventricular ejection fraction; GLS, global longitudinal strain, LDL-C, low-density lipoprotein cholesterol; HbA1c, glycosylated hemoglobin; LVEDVi, left ventricular end-diastolic volume index; LVMi, left ventricular mass index; IVSd, interventricular septum diameter; LVPWd, left ventricular posterior wall diameter; RWT, relative wall thickness;*

* *p < 0.05;*

** *p < 0.01;*

*** *p < 0.001*.

† *Multiple adjustment includes: sex, age, BMI, LVEF, GLS, heart rate, LDL, HbA1c, hypertension, LVEDVi, LVMi*.

**Table 4:** Echocardiographic patterns in participants with LV normal geometry according to the presence of hypertension.

	**Total sample**	**Without hypertension**	**With hypertension**	***p***
*N* (%)	1,789	1,054 (59)	735 (41)	
Women	879 (49)	572 (54)	307 (42)	<0.001
Age, years	53 (12)	49 (10)	59 (10)	<0.001
BSA [m^2^]	1.9 (0.2)	1.87 (0.21)	1.94 (0.23)	<0.001
BMI [kg/m^2^]	26 (4)	25 (4)	27 (4)	<0.001
SBP [mmHg]	130 (17)	121 (11)	142 (17)	<0.001
DBP [mmHg]	78 (10)	75 (7)	83 (10)	<0.001
LVEF [%]	61 (4)	61 (4)	60 (5)	<0.001
GLS [–%],	21 (3)	21 (4)	20 (2)	<0.001
E prime lateral (cm/s)	11 (3)	12 (3)	10 (3)	<0.001
E prime septal (cm/s)	9 (2)	9 (2)	8 (2)	<0.001
LAV [ml]	46 (15)	43 (14)	50 (17)	<0.001
LAVi [ml/m^2^]	24 (7)	23 (6)	26 (8)	<0.001
LVEDVi [mL/m^2^]	52 (10)	52 (11)	52 (10)	0.256
LVMi [g/m^2^]	71 (15)	67 (13)	76 (15)	<0.001
GCW [mmHg%]	2,501 (424)	2,372 (310)	2,687 (491)	<0.001
GWW [mmHg%]	81 (58, 118)	74 (53, 100)	97 (67, 136)	<0.001
GWI [mmHg%]	2,276 (392)	2,167 (294)	2,431 (457)	<0.001
GWE [%]	96 (95, 97)	96 (95, 97)	96 (94, 97)	<0.001

*Data are n (%), mean (SD), or median (quartiles)*.

*BSA, body surface area; BMI, body mass index; SBP, systolic blood pressure; DBP, diastolic blood pressure; LVEF, left ventricular ejection fraction; GLS, global longitudinal strain; LAV, left atrial volume; LAVi, left atrial volume index; LVEDVi, left ventricular end-diastolic volume index; LVMi, left ventricular mass index; GLS, global longitudinal strain; GCW, global constructive work; GWW, global wasted work; GWI, global work index; GWE, global work efficiency*.

**Table 5:** MyW indices in individuals with normal LV geometry according to blood pressure category.

	**Blood pressure categories**					
	**All individuals (*N* = 1,789)**	**Optimal SBP <120 (*N* = 570)**	**Normal SBP 120–129 (*N* = 398)**	**High-normal SBP 130–139 (*N* = 355)**	**Hypertensive SBP ≥140 (*N* = 466)**	***P* for trend**
Women	879 (49)	383 (67)	164 (41)	142 (40)	190 (41)	<0.001
Age [years]	53 (12)	49 (10)	50 (11)	56 (11)	59 (10)	<0.001
LVEF [%]	61 (4)	61 (4)	61 (4)	60 (4)	60 (5)	0.010
GLPS [–%]	21 (3)	22 (5)	21 (3)*	20 (2)	20 (3)	<0.001
SBP [mmHg]	130 (17)	112 (7)	125 (3)	134 (3)	152 (12)	<0.001
GCW [mmHg%]	2,501 (424)	2,224 (276)	2,406 (302)	2,545 (299)	2,888 (444)	<0.001
GWW [mmHg%]	81 (58, 118)	68 (49, 92)	77 (55, 110)	87 (62, 120)	105 (77, 149)	<0.001
GWI [mmHg%]	2,276 (392)	2,038 (267)	2,193 (286)	2,310 (289)	2,611 (425)	<0.001
GWE [%]	96 (95, 97)	96 (95, 97)	96 (95, 97)	96 (95, 97)	96 (94, 97)	<0.001
LVMi [g/m^2^]	71 (15)	65 (14)	70 (14)	73 (14)	77 (15)	<0.001
LVEDVi [ml/m^2^]	52 (10)	51 (10)	52 (10)	53 (11)	52 (10)	0.056
RWT	0.33 (0.05)	0.31 (0.05)	0.32 (0.05)	0.33 (0.04)	0.34 (0.05)	<0.001

*Data are n (%), mean (SD), or median (quartiles)*.

*P for trend (Jonckheere Terpstra trend test and Chi-square test, as appropriate)*.

*BP, blood pressure; LVEF, left ventricular ejection fraction; GLS, global longitudinal strain; SBP, systolic blood pressure, GCW, global constructive work, GWW, global wasted work; GWI, global work index; GWE, global work efficiency; LVMi, left ventricular mass index; LVEDVi, left ventricular end-diastolic volume index; RWT, relative wall thickness*.

## Discussion

The current study investigated the association of altered LV geometry with MyW indices in a large, population-based sample. Three major findings emerged. First, while the majority of individuals studied exhibited a normal LV geometry, a relevant proportion of participants revealed an abnormal LV geometry; these subjects were older and presented with a less favorable profile of cardiovascular risk factors. Second, both LV enlargement and LV hypertrophy were adversely associated with GWE, predominantly through increasing the amount of GWW. Third, when compared to participants without hypertension, individuals with normal LV geometry and concomitant hypertension exhibited larger LV mass and LA volume and less favorable measures of systolic and diastolic function. Their MyW pattern was characterized by higher GCW and GWW and thus lower GWE, comparable to the pattern found in LV hypertrophy.

Altered LV geometry, including its components LV mass and LV volume, constitute pivotal information of the standard echocardiography report (1), as they reliably indicate maladaptation due to adversely regulated hemodynamics (22). Such conditions trigger myocardial responses that aim at maintaining a normal cardiac output despite compromised energetics (23–25). When left untreated, these adaptive changes induce early, subclinical changes in LV geometry, advance toward subclinical impairment in LV function (1), and ultimately cause functional capacity loss (26). This complex configuration is mainly driven by changes at the histological and metabolic level, e.g., myocyte hypertrophy, apoptosis, and energy consumption (27). Not surprisingly, deteriorating LV geometry was shown to predict incident heart failure (28, 29).

An increased hemodynamic load, induced either by pressure, e.g., in hypertension, or by volume, e.g., in valvular disease, or by a combination of both stimuli, contributes to LV hypertrophy and/or dilation, resulting in different geometric adaptations (1, 2). Recently, changes in LV chamber geometry, i.e., an increase in LV mass and/or LV size, were reported to relate to impaired GLS (30). LV mass and LV volume further impact on electric conduction times resulting in prolonged QRS duration and potential consecutive LV dyssynchrony (31–34), which, in turn, is also known to adversely affect GLS (30, 35). Echocardiography-based determination of MyW parameters now offers the possibility to non-invasively study the different components of active myocardial function and to apply this method to larger collectives. Covering both the impairment of longitudinal LV function and a potential LV dyssynchrony induced by conduction delays, MyW might advance our mechanistic understanding of the myocardial function and subsequent adaptive changes in individuals with abnormal LV geometry. We determined three pathological groups (see Figure 1), which serve as examples of a (well-acknowledged) disease paradigm characterizing the gradual alteration of LV morphology over time given certain risk constellations (1, 19).

### Concentric LV Remodeling and Concentric Hypertrophy

CR dominated in our study sample, followed by EH and CH. CR is considered a late-stage response of the LV to adverse hemodynamic circumstances and is predominantly caused by pressure overload as induced by increased afterload (36) due to arterial hypertension or aortic stenosis (37), or volume overload (1). CR is associated with adverse LV function (38, 39) and an adverse prognosis when compared to normal LV geometry (4, 40, 41). In our sample, participants with CR were older and showed a less favorable risk factor and comorbidity profile and lower values of GLS when compared to participants with normal LV geometry. The more detailed analysis of LV myocardial function revealed a trend toward an increase in GCW and GWI (Figure 1), which might be a consequence of increased myocardial muscle power in LV hypertrophy, and was even more pronounced in CH. In addition to this increase in constructive myocardial work, participants with CR and CH exhibited significantly higher levels of GWW when compared to participants with normal LV geometry. The lower values of global work efficiency suggest that the proportionate increase in GWW exceeds the increase in GCW with progressing LV hypertrophy might be one explanation for impaired exercise capacity in individuals with LV hypertrophy and abnormal LV geometry (42). Further, these findings were even more pronounced in individuals with CH. As this subgroup was small in our study sample, we did not perform further statistical analyses. However, the CH pattern is of high clinical relevance, and further dedicated studies in hypertensive patients need to provide additional insights.

Arterial hypertension is one of the most prevalent cardiovascular risk factors and a major contributor to long-term changes in LV geometry (36, 37, 43, 44). A higher prevalence of hypertension was seen with a deviation from normal LV geometry. However, even in participants with measures of LV geometry within a normal range, we found notable differences in LV structure and function in individuals with and without hypertension. Among subjects with normal LV geometry, those with hypertension presented with equal LV size but with higher LV mass when compared to subjects without hypertension (Table 4). The LV myocardium of those with hypertension performed a higher amount of work, constructive (GWI, GCW) and wasted work, at a lower efficiency level. A detailed analysis of LV structure and function according to the current blood pressure during the study visit showed a similar pattern (Table 5). Higher SBP values were associated with higher LV mass though still within the normal range. Participants with normal and high-normal BP had higher LV mass when compared to participants with optimal BP. Further, normal and high-normal blood pressure were associated with significantly higher values of work performed by the myocardium, including wasted work, when compared to optimal blood pressure (Table 5). As part of the adaptation process, it appears that the LV hypertrophies to perform a higher amount of work. Due to a disproportionate increase in wasted work, work efficiency seems to be affected already in individuals with high-normal blood pressure, hence in a very early stage of disease (Table 5). Our results give a glimpse of mechanistic insights into the pathophysiology of hypertensive heart disease and highlight the importance of early and consistent treatment of arterial hypertension to reach optimal treatment goals.

### Eccentric Hypertrophy

This phenotype is characterized by increased LV size (i.e., LV dilatation) in the presence of normal wall thickness. EH is typically found in states of chronic volume overload, such as significant mitral regurgitation (which was excluded from our study sample), but also as an early manifestation of a cardiomyopathic process (1, 36). Further, previous work from our population-based cohort reported a higher proportion of increased LV volumes in individuals with structural heart disease with no clinical HF symptoms and absent CV risk factors known as the B-not-A group of HF (16). Participants with EH were older, more often female, had higher NT-proBNP levels, and a higher prevalence of hypertension, diabetes, and dyslipidemia when compared to normal LV geometry or CR. GCW and GWI were normal among individuals with EH, but GWW was markedly enhanced and GWE compromised. Of note, GWW and GWE were predominantly determined by larger LV volumes, potentially as a consequence of increased wall stress in larger LV volumes (45). Our results extend first analyses from NORRE (46), a multinational study to generate normal values for echocardiographic measures, where mild univariate associations between LV size and MyW indices were found that vanished in multivariable analysis, possibly due to issues of sample size and selection criteria. In contrast to a concentric increase in LV mass, an increase in LV size without an increase in LV wall thickness seems to be associated with an increase in GWW only, and lower GWE.

Cardiomyopathies are characterized by heterogeneous patterns of LV hypertrophy and progressive LV enlargement leading to myocardial dysfunction (47–49) and, on a histological level, by cardiomyocyte hypertrophy, myocardial disarray, and interstitial fibrosis (49). Recent work in patients with cardiomyopathy showed impaired MyW indices when compared to healthy controls (48, 50, 51). MyW analysis was hypothesized to reveal the effect of chronic remodeling on myocardial function in patients with cardiomyopathies, unmasking, i.e., a low capacity to adjust to an increased workload (52). Chan et al. (50) suggested that wasted work may be related to the increased myocardial wall stress against a higher afterload. Likewise, wasted work results to be of great interest as a potential factor reducing LV work efficiency and ultimately might contribute to LV remodeling. LV remodeling and consecutive functional changes reflect myocardial glucose metabolism and energetics (53), which was shown to correlate with non-invasive echocardiography-derived MyW indices (8). Our results show additional insights into the relationship of LV mass and size with myocardial work and might contribute to the elucidation of pathophysiological processes in cardiomyopathies.

### Limitations and Strengths

In this large population-based sample, cardiovascular risk factors were comprehensively and carefully assessed according to standard operating procedures. In particular, echocardiography was performed by well-trained and internally certified and quality-controlled sonographers (18). However, the current cross-sectional analysis cannot inform on longitudinal alterations and causal inferences. The size of the three subgroups emerging with an abnormal LV geometry was relatively small. Nevertheless, due to the representative mode of sampling, they mirror the frequencies of these abnormalities in the population free of heart failure. For the derivation of MyW parameters, ideally, blood pressure should be measured during the echocardiographic examination. In STAAB participants, blood pressure was measured in a sitting position after 5 min of rest in a separate room but immediately prior to the echocardiographic examination. Hence, the thus introduced imprecision is likely to be small. Technically and physiologically, information on MyW should not be regarded as the exact equivalent to investigations on pressure-volume loop recordings (10, 45, 54). As discussed elsewhere in detail, MyW does not account for radial, and circumferential LV function nor wall stress since LV radial curvature and wall thickness are not part of its derivation from pressure-strain loops (45, 54). Comparison of MyW, particularly of GWW, between different hearts, however, is considered a valid measure since it is a relative measure that compensates for limited information about local geometry and consecutive potential differences in wall stress (10). Further, MyW integrates LV systolic longitudinal strain, blood pressure, and time intervals, thus comprehensively accounting for potential impairment (a) in LV longitudinal contraction and (b) and in cardiac conduction induced by abnormal LV geometry as apparent, e.g., in patients with heart failure.

## Conclusion

MyW analysis is a non-invasive, echocardiography-based method facilitating new insights into the relationship of LV geometry and myocardial performance in this population-based cohort free from heart failure. Any deviation from a normal LV geometric profile was associated with an alteration of MyW. While LV dilation was associated with solely higher GWW, concentric remodeling and hypertrophy were associated with both higher GCW and GWW. A disproportionately higher GWW resulted in lower GWE. These altered MyW patterns were already present in hypertensive individuals with normal LV geometry and might thus serve as an early sign of incipient hypertensive heart disease. Longitudinal studies are needed to test this hypothesis and improve our understanding on the mechanisms of hypertensive heart disease and the time course of its evolvement.

## Data Availability Statement

The raw data supporting the conclusions of this article will be made available by the authors, without undue reservation.

## Ethics Statement

The studies involving human participants were reviewed and approved by Ethics Committee of the Faculty of Medicine, University of Würzburg (vote #98/13) and data protection officer of the University of Würzburg (#J-117.605-09/13). The patients/participants provided their written informed consent to participate in this study.

## Author Contributions

FS, CM, GG, PH, and SS concepted and designed the study. FS, CM, VC, JA, FE, GG, PH, and SS analysis and interpretation of data. FS drafted the manuscript. CM, VC, JA, FE, GG, PH, and SS revised the manuscript critically for intellectual content. All authors contributed to the article and approved the submitted version.

## Conflict of Interest

The authors declare that the research was conducted in the absence of any commercial or financial relationships that could be construed as a potential conflict of interest.

## Abbreviations

LV: left ventricle/ventricular
LVMi: left ventricular mass index
LVEDVi: left ventricular end diastolic volume index
CR: concentric remodeling
CH: concentric hypertrophy
EH: eccentric hypertrophy
GLS: global longitudinal strain
GWE: global work efficiency
GWI: global work index
GCW: global constructive work
GWW: global wasted work
STAAB: The Characteristics and Course of Heart Failure STAges A/B and Determinants of Progression Cohort Study.
